# N^6^-Methyladenosine RNA Modification in Host Cells Regulates Peste des Petits Ruminants Virus Replication

**DOI:** 10.1128/spectrum.02666-22

**Published:** 2023-02-14

**Authors:** Owais Khan, Gunturu Narasimha Tanuj, Divyaprakash R. Choravada, Kaushal Kishore Rajak, S Chandra Sekar, Madhu Cholenahalli Lingaraju, Sujoy K. Dhara, Praveen K. Gupta, Bishnu Prasad Mishra, Triveni Dutt, Ravi Kumar Gandham, Basavaraj Sajjanar

**Affiliations:** a Veterinary Biotechnology Division, ICAR—Indian Veterinary Research Institute, Izatnagar Bareilly, Uttar Pradesh, India; b Biological Products Division, ICAR—Indian Veterinary Research Institute, Izatnagar Bareilly, Uttar Pradesh, India; c Division of Virology, ICAR—Indian Veterinary Research Institute, Mukteshwar, Uttarakhand, India; d Pharmacology and Toxicology Division, ICAR—Indian Veterinary Research Institute, Izatnagar Bareilly, Uttar Pradesh, India; e ICAR—National Bureau of Animal Genetic Resources, Karnal, Haryana, India; Wayne State University

**Keywords:** RNA modifications, N^6^-methyladenosine (m^6^A), small ruminant morbillivirus, virus replication, virus-host interactions

## Abstract

N^6^-methyladenosine (m^6^A) modification is a major RNA epigenetic regulatory mechanism. The dynamics of m^6^A levels in viral genomic RNA and their mRNAs have been shown to have either pro- or antiviral functions, and therefore, m^6^A modifications influence virus-host interactions. Currently, no reports are available on the effect of m^6^A modifications in the genome of *Peste des petits ruminants virus* (PPRV). In the present study, we took PPRV as a model for nonsegmented negative-sense single-stranded RNA viruses and elucidate the role of m^6^A modification on viral replication. We detected m^6^A-modified sites in the mRNA of the virus and host cells, as well as the PPRV RNA genome. Further, it was found that the level of m^6^A modification in host cells alters the viral gene expression. Knockdown of the METTL3 and FTO genes (encoding the m^6^A RNA modification writer and eraser proteins, respectively) results in alterations of the levels of m^6^A RNA modifications in the host cells. Experiments using these genetically modified clones of host cells infected with PPRV revealed that both higher and lower m^6^A RNA modification in the host cells negatively affect PPRV replication. We found that m^6^A-modified viral transcripts had better stability and translation efficiency compared to the unmodified mRNA. Altogether, from these data, we conclude that the m^6^A modification of RNA regulates PPRV replication. These findings contribute toward a way forward for developing novel antiviral strategies against PPRV by modulating the dynamics of host m^6^A RNA modification.

**IMPORTANCE** Peste des petits ruminants virus (PPRV) causes a severe disease in sheep and goats. PPRV infection is a major problem, causing significant economic losses to small ruminant farmers in regions of endemicity. N^6^-methyladenosine (m^6^A) is an important RNA modification involved in various functions, including virus-host interactions. In the present study, we used stable clones of Vero cells, having knocked down the genes encoding proteins involved in dynamic changes of the levels of m^6^A modification. We also used small-molecule compounds that interfere with m^6^A methylation. This resulted in a platform of host cells with various degrees of m^6^A RNA modification. The host cells with these different microenvironments were useful for studying the effect of m^6^A RNA modification on the expression of viral genes and viral replication. The results pinpoint the level of m^6^A modifications that facilitate the maximum replication of PPRV. These findings will be useful in increasing the virus titers in cultured cells needed for the economical development of the vaccine. Furthermore, the findings have guiding significance for the development of novel antiviral strategies for limiting PPRV replication in infected animals.

## INTRODUCTION

As obligatory parasites, viruses utilize the metabolic and protein synthesis machinery of the host cells, eventually prioritizing their own multiplication (1–3). Most of the events during virus-host interactions are markedly influenced by different gene regulatory mechanisms (4). Recently, it was reported that modifications in the nucleotide base of mRNA have important cellular functions. N^6^-methyladenosine (m^6^A) is one such modification that regulates gene expression by modulating transport, processing, translation, and decay of mRNA (5). The m^6^A modifications (addition of a methyl group at the N^6^ position of the adenosine base) are catalyzed by writer proteins—methyltransferases (METTL3, METTL14, and WTAP)—and removed by eraser proteins—demethylases (FTO and ALKBH5) (6). The regulatory functions of m^6^A modification are mediated by readers—YTH domain-containing proteins (YTHDF1, YTHDF2, YTHDF3, YTHDC1, and YTHDC2) (7). The reversible and dynamic nature of m^6^A modification in host cells indicates that it may play a potential role in regulating the viral replication and the outcome of viral infections (8).

Viral infections cause changes in the m^6^A modification machinery of host cells, and the level of m^6^A modification within the host cells may influence viral replication (9). According to viral epitranscriptomic studies, m^6^A modification can have either pro- or antiviral functions, depending on the nature of the virus (different types of virus life cycles) (10–12). In the case of the DNA virus Kaposi’s sarcoma-associated herpesvirus (KSHV), lytic replication is regulated by m^6^A modification of a key lytic switch protein called RTA (replication transcription activator), and the level of m^6^A modification determines the RTA pre-mRNA splicing. In addition, it has also been noted that KSHV employs a smart mechanism to manipulate the host m^6^A machinery in promoting lytic replication (13). In retroviruses, HIV-1 was found to increase the m^6^A modification in the mRNA fraction of infected T cells. Furthermore, knockdown of m^6^A writers (METTL3/METTL14) and erasers (ALKBH5) in the host cells affects the expression of viral env and p24 capsid proteins (14). In addition, m^6^A-modified sites are clustered in the HIV-1 3′ untranslated region (3′ UTR) and mediate enhanced viral gene expression and viral replication through a mechanism involving reader proteins (15). For example, cellular YTHDF reader proteins bind to m^6^A-modified sites and facilitate viral gene expression and replication in CD4^+^ T cells (16). Similarly, murine leukemia virus (MLV) RNA bears m^6^A modifications, and mutational removal of modification sites (or altered expression of m^6^A factors) affected MLV replication (17).

Different members of flaviviruses (positive-sense single-stranded RNA viruses) such as Zika virus (ZIKV), dengue virus (DENV), West Nile virus (WNV), and hepatitis C virus (HCV) were found to contain m^6^A-modified sites that play an important role in viral gene expression and replication (18). Reduced m^6^A levels through knockout of METTL3 and METTL14 increased HCV replication. The negative correlations between m^6^A levels and viral replication were in contrast and opposite to a phenomenon observed earlier with retroviruses. In human lung epithelial cells, inactivation of METTL3 due to mutation inhibited the replication of the influenza A virus (IAV), a segmented RNA virus. Even though m^6^A was found to be advantageous for the IAV life cycle, elevated m^6^A levels were not observed in all viral RNAs, including mRNA/cRNA (plus) strands and viral RNA (vRNA) (minus) strands (19). Further, higher m^6^A modification facilitated viral gene expression in multiple viruses, such as SV-40 virus, respiratory syncytial virus (RSV), human metapneumovirus (HMPV), and herpesvirus type 1 (HSV-1) (20–23). In recent publications, m^6^A was reported to promote replication of severe acute respiratory syndrome coronavirus 2 (SARS-CoV-2) (24, 25). Alteration in m^6^A in flaviviruses also affects transcription in host cells (18). In another interesting report, m^6^A modification was found to increase the stability of HIV-1 transcripts, but simultaneously, in the same cell, it exerted exactly the opposite effect, decreasing the stability of host mRNAs (26). These results reflect that m^6^A modification may be sequence specific and dependent on the origin of the transcript, whether from the host or from the invading virus. Taken together, the results also indicate that m^6^A-mediated effects are not uniform and appear to depend on the nature of viruses and differences in their life cycle stages. The mechanistic details of the m^6^A-mediated regulation of viral replication need to be understood separately for important pathogenic viruses.

In the present study, we used small ruminant morbillivirus, or peste des petits ruminants virus (PPRV), as a model for nonsegmented negative-sense single-stranded RNA viruses belonging to the family *Paramyxoviridae*. PPRV causes an acute, highly contagious viral disease in sheep and goats, leading to severe economic losses for small ruminant farmers in regions of endemicity.

Here, we report the role of m^6^A RNA modifications in the PPRV-host interaction. We show that PPRV infection affects the levels of m^6^A modification of the host cells. The infection also alters the expression of m^6^A writer (METTL3 and WTAP) and eraser (FTO and ALKBH5) proteins. The reduction of m^6^A modification in host cells using 3-deazaadenosine (3-DAA) decreases PPRV replication. Further, we demonstrate that knockdown of writer (METTL3) and eraser (FTO) genes affects PPRV replication in a stable clone of host cells. Taken together, our results indicate that PPRV replication is highest in host cells, with relatively optimum m^6^A RNA modifications. Finally, we show that the presence of m^6^A in viral mRNA facilitates both stability and translation efficiency for better PPRV gene expression, and these events contribute to viral replication.

## RESULTS

### PPRV contains m^6^A modifications in its RNA.

**FIG 1:**
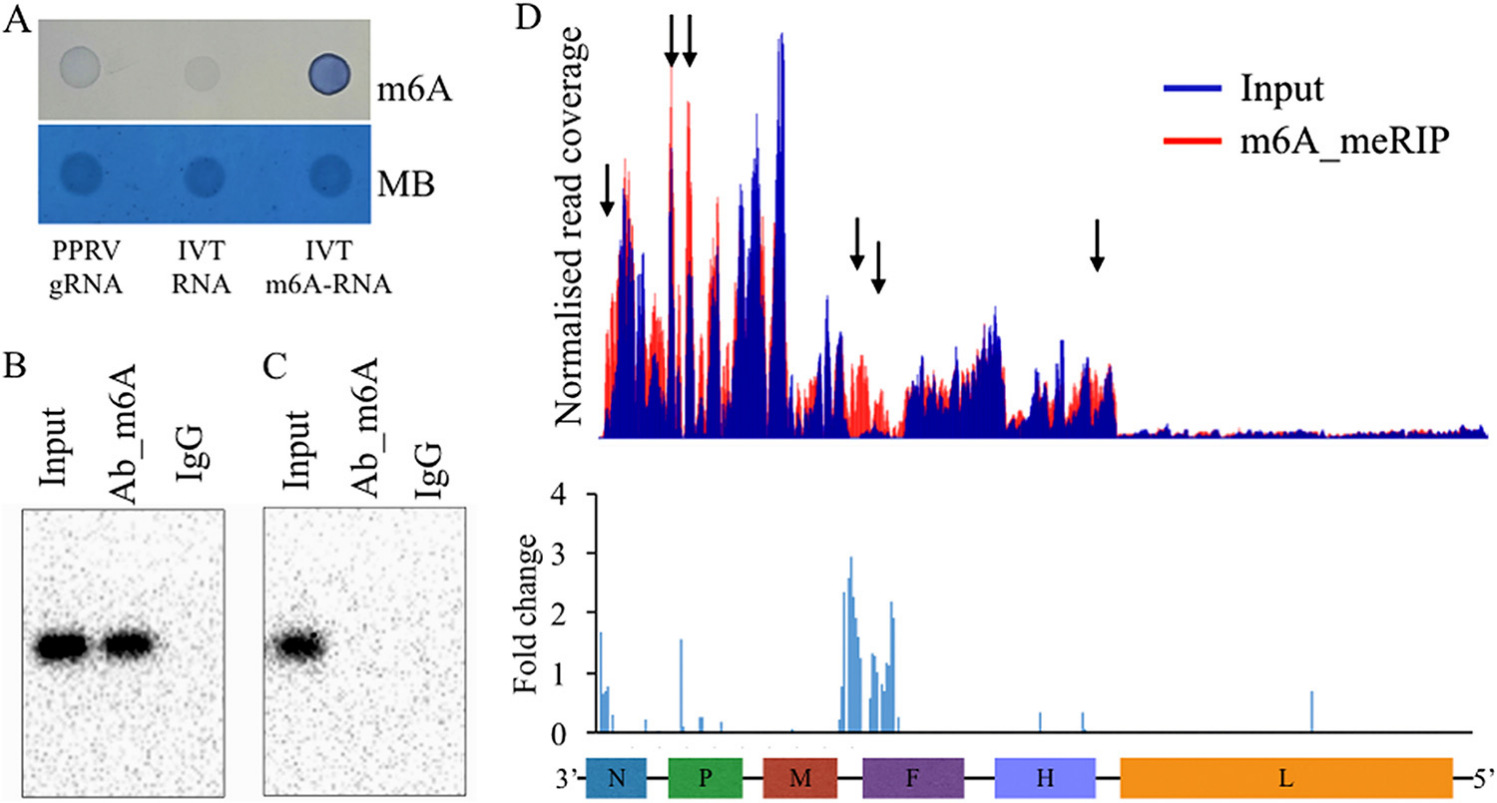
PPRV genomic RNA (gRNA) and mRNA transcripts contain m^6^A modifications. (A) Dot blot assay for PPRV genomic RNA obtained from ultrapurified virus and *in vitro* T7-transcribed (IVT) RNA (with or without m^6^A) detected with m^6^A-specific antibody, using MB (methylene blue) as the loading control. (B) MeRIP-Northern blotting for mRNA from PPRV-infected Vero cells. (C) MeRIP-Northern blotting for RNA prepared from IVT PPRV detected with IgG (control) or m^6^A-specific antibody. (D) MeRIP-Seq for total RNA extracted from PPRV-infected Vero cells and subjected to immunoprecipitation with m^6^A-specific antibody, followed by next-generation sequencing. Methylation coverage on the full-length input RNA and m^6^A_MeRIP are presented, along with the fold change.

PPRV genomic RNA isolated from ultrapurified virus showed the presence of m^6^A modifications in the dot blot assay (Fig. 1A). Identification of m^6^A modifications in the PPRV transcripts was performed using Northern blot and m^6^A-meRIP (methylated RNA immunoprecipitation) sequencing. Here, we used RNA from two sources: (i) total RNA from PPRV-infected Vero cells and (ii) *in vitro* T7-transcribed (IVT) PPRV RNA. In the Northern blot, both the input and m^6^A antibody-enriched RNA originating from the PPRV-infected Vero cells displayed the presence of m^6^A modifications. However, only the input RNA but not the m^6^A antibody-enriched RNA originating from IVT showed a positive result, as the IVT RNA lacked m^6^A (Fig. 1B and C). Methylated RNA immunoprecipitation sequencing (MeRIP-Seq) data analysis revealed the presence of m^6^A-modified regions in the viral mRNAs. Six m^6^A peaks were identified across different viral genes. The highest fold change was found in the region where the viral matrix (M) and fusion protein (F) genes are located. Other peaks were observed in the coding regions of the nucleocapsid (N) and phosphoprotein (P) genes (Fig. 1D). Overall, these results indicate that PPRV contains m^6^A-modified nucleotides in their mRNA and genomic RNA.

### PPRV infection affects the level of m^6^A modification and its related proteins.

**FIG 2:**
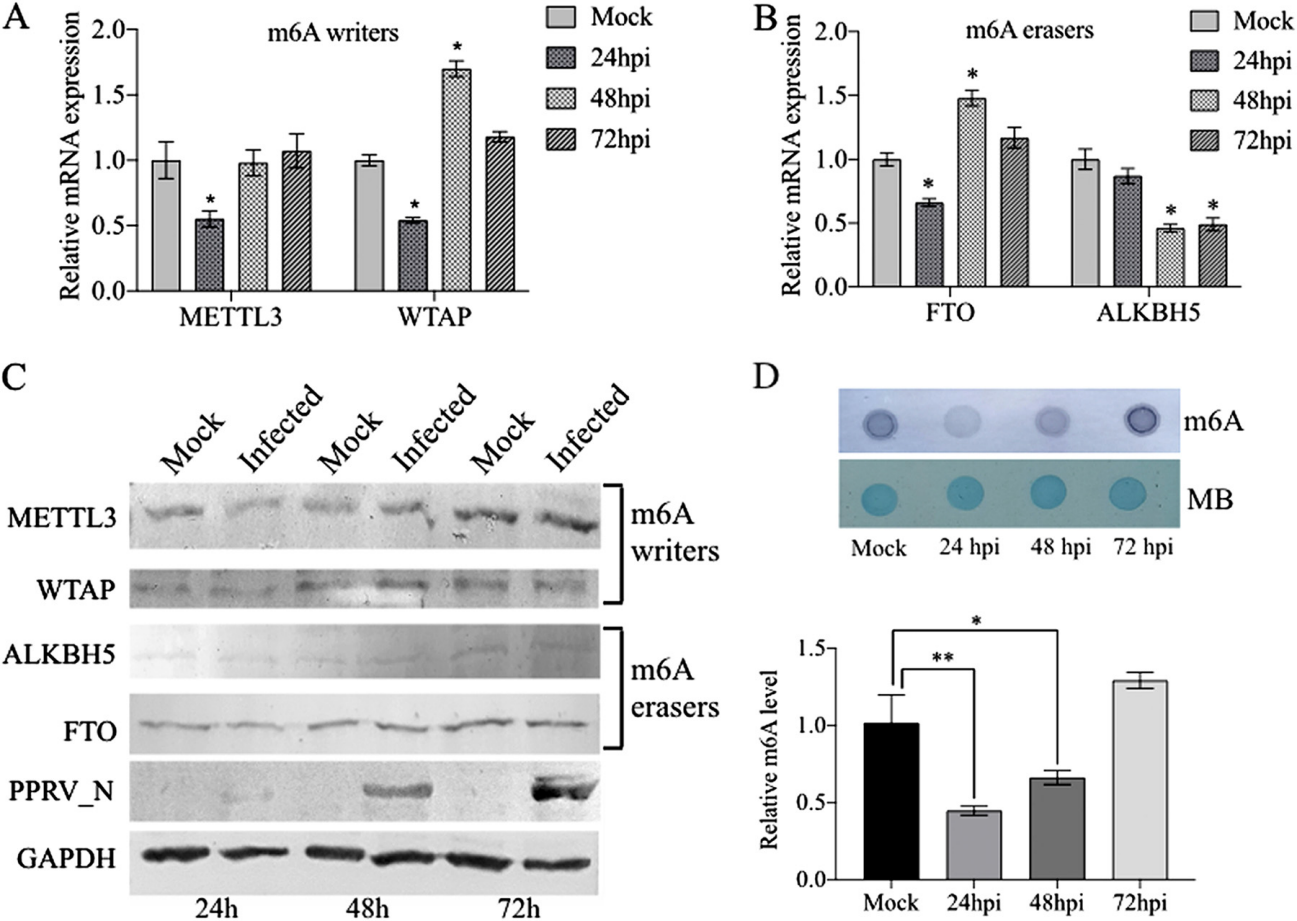
Effect of PPRV infection on expression of m^6^A modification proteins and m^6^A levels. Vero cells were infected with PPRV for different lengths of time (24, 48, and 72 h). The expression of m^6^A machinery genes was evaluated by RT-qPCR, proteins by Western blotting (WB), and m^6^A levels by dot blot. (A) Expression of m^6^A writers (METTL3 and WTAP). (B) Expression of m^6^A erasers (FTO and ALKBH5). (C) WB for m^6^A writers and eraser proteins. (D) Effect of PPRV infection on the level of m^6^A modification in the mRNA of host cells and MB (methylene blue) as the loading control. Error bars indicate the standard deviations; *, *P* < 0.05.

To understand the effect of PPRV infection on host m^6^A modification, we analyzed PPRV-infected host cells for m^6^A levels and also for the expression of genes related to m^6^A modification. It was found that there was reduced expression of m^6^A reader proteins (METTL3 and WTAP) at 24 h postinfection (hpi). We also observed similar changes in the m^6^A eraser protein (FTO). Further, expression of WTAP and FTO was found to have increased at 48 hpi. (Fig. 2A and B). PPRV infection did not cause similar patterns of changes to the protein levels of these genes as observed in reverse transcription-quantitative PCR (RT-qPCR). The changes observed in the protein levels were not significant. Expectedly, the viral nucleocapsid (N) protein significantly increased at 48 hpi and 72 hpi (Fig. 2C), indicating the establishment and temporal progression of PPRV infection in the host cells. The m^6^A levels in the PPRV-infected host cells decreased at 24 hpi, followed by a subsequent increase, with the highest levels attained at 72 hpi. The phenomenon was accompanied by the expected patterns of m^6^A writer and eraser gene expression (Fig. 2D).

**FIG 3:**
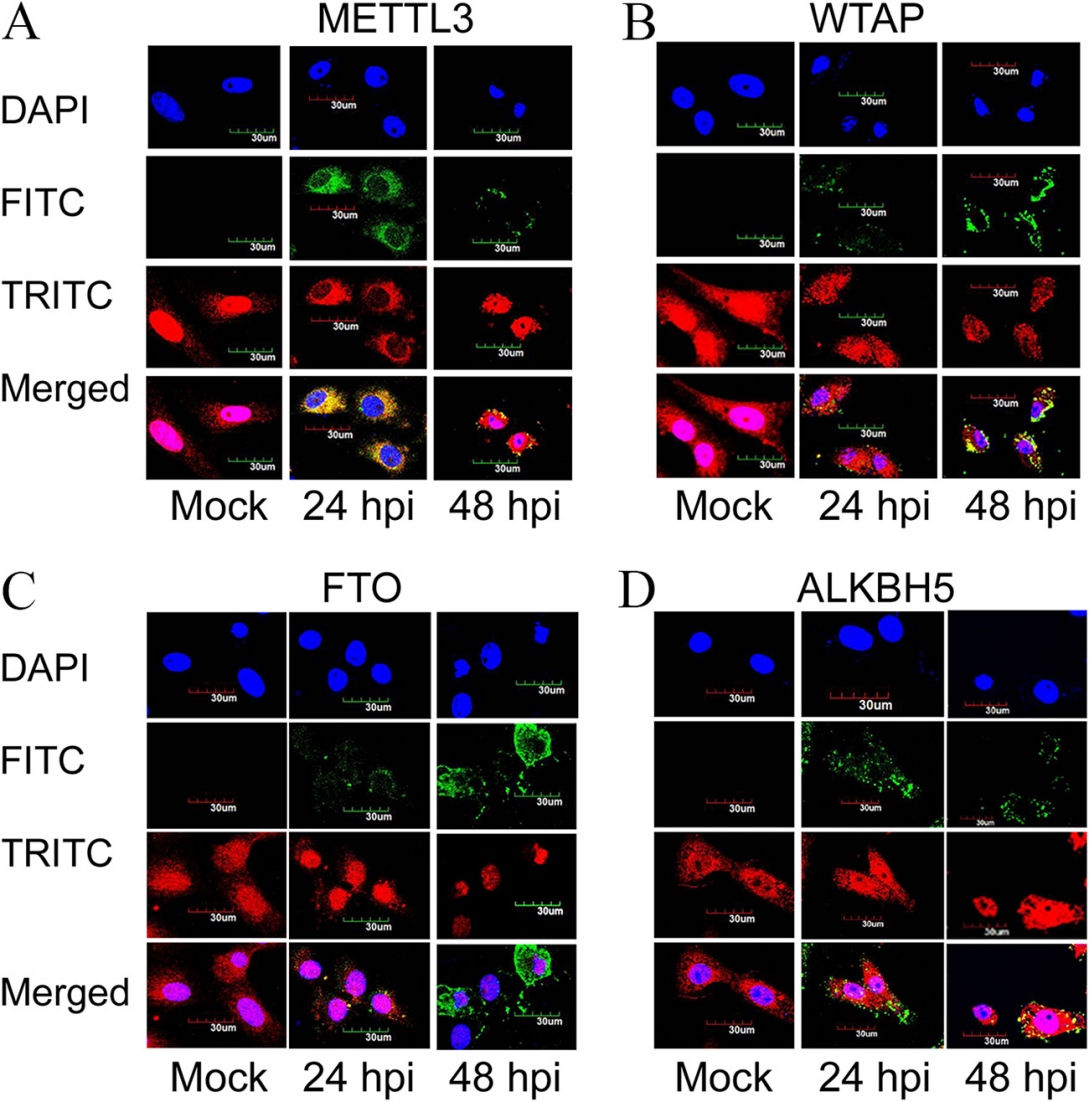
Effect of PPRV infection on the distribution of m^6^A machinery proteins and their colocalization with PPRV nucleocapsid (N) protein. (A and B) m^6^A writer proteins METTL3 (A) and WTAP (B). (C and D) m^6^A eraser proteins FTO (C) and ALKBH5 (D). These m^6^A machinery proteins were analyzed along with the PPRV nucleocapsid (N) protein at different time points of infection by double immunofluorescence staining using laser scanning confocal microscopy. Viral nucleocapsid (N) protein is stained green (FITC [fluorescein isothiocyanate]), host m^6^A modification proteins are stained red (TRITC [tetramethyl rhodamine isocyanate]), and the nucleus is stained blue (DAPI [4′,6-diamidino-2-phenylindole]).

We performed double immunofluorescence staining for PPRV nucleocapsid (N) and m^6^A machinery proteins with specific antibodies at 24 and 48 hpi. METTL3 was found more in the nucleus in uninfected cells. However, it was mainly redistributed to the cytoplasm at 24 hpi, and at 48 hpi, it was found equally in the nucleus and the cytoplasm. WTAP was found in both the cytoplasm and the nucleus in uninfected cells and remained the same in the virus-infected cells (at both 24 hpi and 48 hpi). In the case of FTO, the protein was found in both the nucleus and the cytoplasm of uninfected cells. However, it was redistributed to the nucleus after infection (24 hpi and 48 hpi). ALKBH5 protein was found distributed in both the nucleus and the cytoplasm in uninfected cells, and no changes in distribution were observed after infection (Fig. 3).

### Inhibition of m^6^A modification reduces PPRV replication.

**FIG 4:**
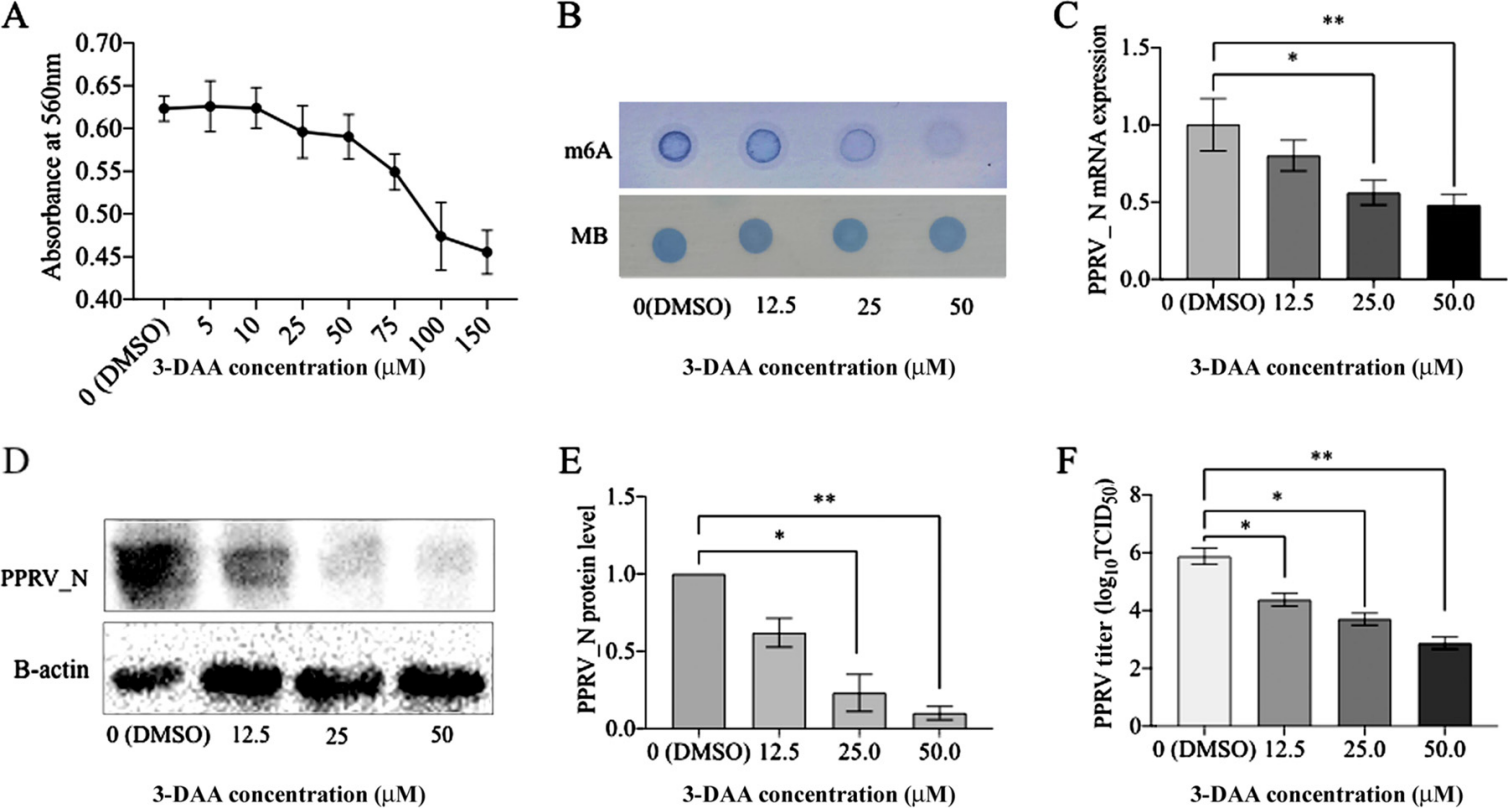
Effect of reduction in modified m^6^A in host cells on PPRV gene expression and replication. (A) Effect of 3-DAA on the proliferation of Vero cells, evaluated by MTT [3-(4,5-dimethyl-2-thiazolyl)-2,5-diphenyl-2H-tetrazolium bromide] assay. DMSO, dimethyl sulfoxide. (B) Dose-dependent reduction of host m^6^A levels by 3-DAA treatment in Vero cell mRNA, confirmed by dot blot assay. Vero cells were treated with 3-DAA and infected with PPRV. The effect of reducing the host m^6^A modifications on PRV gene expression and viral replication was analyzed; MB (methylene blue) was used as the loading control. (C) PPRV nucleocapsid (N) gene expression determined by RT-qPCR. (D and E) PPRV N protein levels determined by Western blotting. (F) Culture supernatant was evaluated for PPRV titration using the TCID_50_ method. Error bars indicate standard deviations; *, *P* < 0.05; **, *P* < 0.01.

A previous report indicated that 3-deazaadenosine (3-DAA) reduces m^6^A modification by decreasing the formation of SAM (S-adenosyl methionine) (23). Initially, the maximum concentration of 3-DAA (50 μM) with no significant impact on the proliferation of cultured Vero cells was determined (Fig. 4A). Later, we confirmed that the m^6^A levels in host cells progressively decreased as the concentration of 3-DAA in the cell culture was increased (Fig. 4B). We treated the cultured Vero cells with 3-DAA, followed by infection with PPRV, and analyzed the viral gene expression and replication. It was found that the expression of PPRV nucleocapsid protein progressively decreased in 3-DAA-treated cells (Fig. 4C to 4E), indicating less virus present in these cells. The PPRV titer was also significantly reduced at a 3-DAA concentration of 50 μM (Fig. 4F). Overall, these results indicate that m^6^A modification in host cells is essential for PPRV replication.

### PPRV replication increased with higher m^6^A RNA modifications.

**FIG 5:**
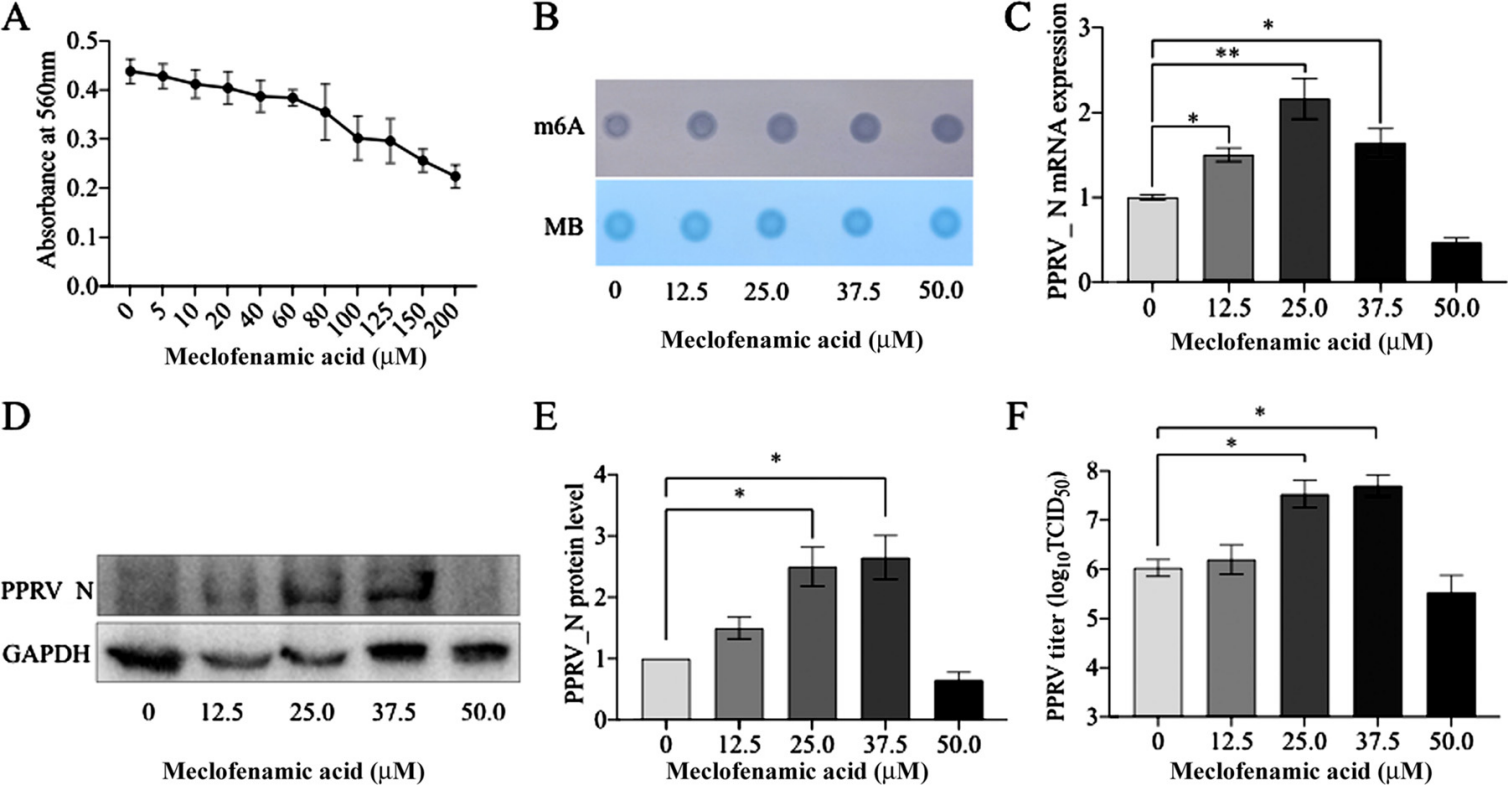
Effect of incremental m^6^A modification in host cells on PPRV gene expression and replication. (A) Effect of meclofenamic acid (MA) on the proliferation of Vero cells as evaluated by MTT assay. (B) Dose-dependent increase in the host m^6^A levels by MA treatment, confirmed by dot blot assay. Vero cells were treated with MA and infected with PPRV. The effect of increased host cell m^6^A modification on PPRV gene expression and viral replication was analyzed; MB (methylene blue) was used as loading control. (C) PPRV nucleocapsid (N) gene expression, determined by RT-qPCR. (D and E) PPRV nucleocapsid (N) protein levels determined by Western blotting. (F) Culture supernatant was evaluated for PPRV titration using the TCID_50_ method. Error bars indicate standard deviations; *, *P* < 0.05; **, *P* < 0.01.

Meclofenamic acid (MA) is a small-molecule inhibitor of FTO (m^6^A eraser protein). For cell culture, we found 60 μM to be the highest concentration of MA that did not significantly affect the proliferation of cultured Vero cells (Fig. 5A). MA treatment increased the levels of m^6^A RNA modification in the host cells in a dose-dependent manner (Fig. 5B). In order to assess the impact of MA treatment on PPRV replication, we treated the cultured Vero cells with MA and then infected them with PPRV. Next, we analyzed the viral gene expression and also quantified the virus titer. We found that MA treatment increased the expression of PPRV nucleocapsid mRNA and protein, with the maximum expression at 37.5 μM MA (Fig. 5C to 5E). However, at a concentration of 50 μM MA, we observed an inhibitory effect. Similar results were also found for PPRV replication, showing higher PPRV titers at 37.5 μM MA and significantly lower PPRV titers after treatment with 50 μM MA (Fig. 5F). MA is also known to inhibit COX enzymes, and its effect on viral replication may be due to COX enzyme inhibition in host cells. To test this possibility, we treated the host cells with a known COX inhibitor (indomethacin), followed by PPRV infection. The results indicated that indomethacin treatment did not significantly alter PPRV gene expression or replication (see Fig. S1 in the supplemental material). Hence, the effect of MA on PPRV gene expression and viral replication was due to its inhibition of FTO (m^6^A eraser) protein, which increased the m^6^A levels in the host cells.

### m^6^A methyltransferase and demethylase regulate PPRV replication.

**FIG 6:**
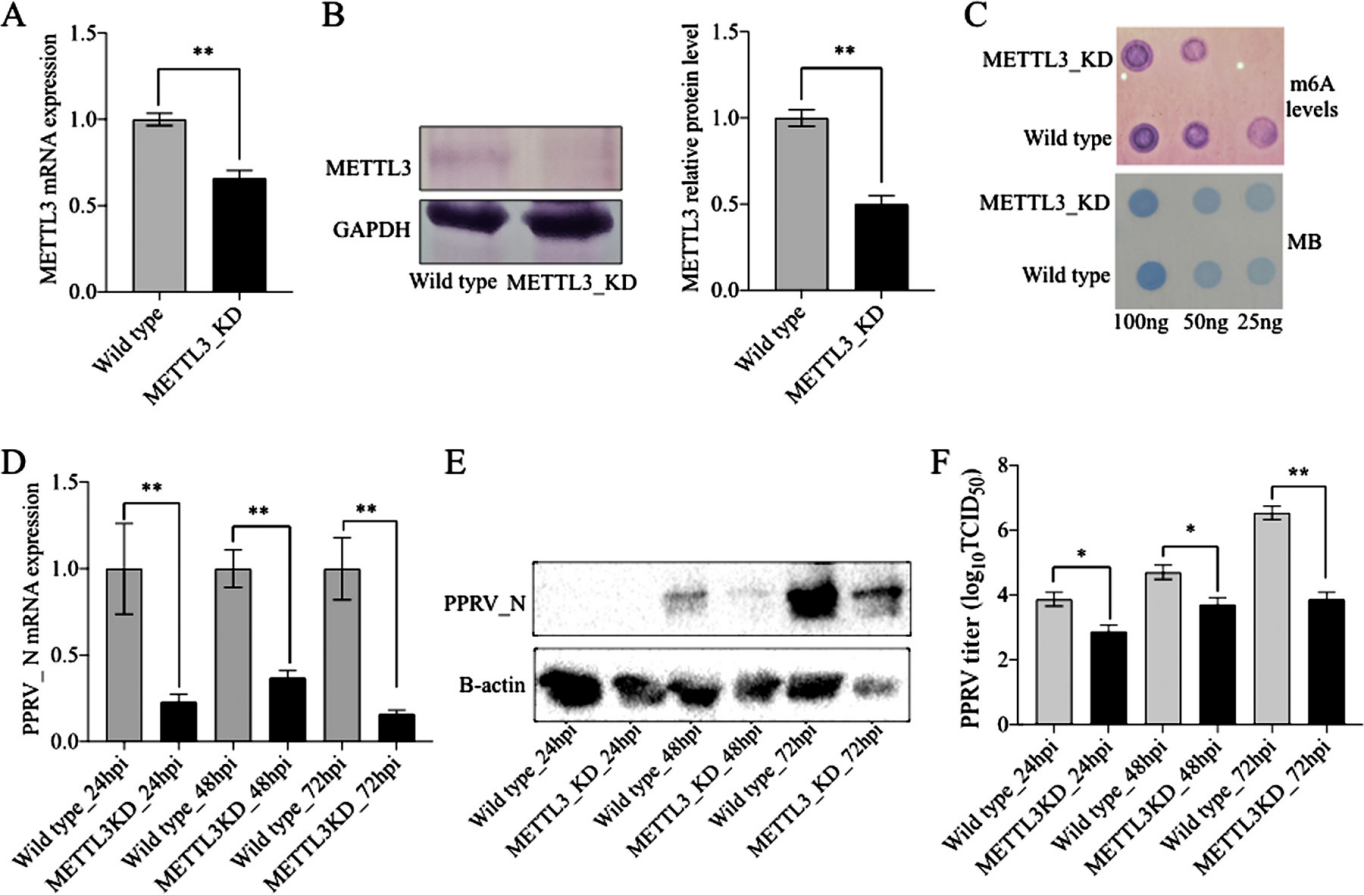
Effect of METTL3 knockdown on PPRV gene expression and replication. (A) METTL3 mRNA expression was analyzed in wild-type and METTL3 stable knockdown (METTL3_KD) Vero cells by RT-qPCR. (B) METTL3 protein level in wild-type and METTL3_KD Vero cells was analyzed by WB. (C) The levels of m^6^A modification in mRNA of wild-type and METTL3_KD Vero cells were analyzed by dot blot assay, with MB (methylene blue) used as the loading control. (D) PPRV nucleocapsid (N) gene expression in wild-type and METTL3_KD Vero cells was analyzed by RT-qPCR. (E) PPRV nucleocapsid protein level in wild-type and METTL3_KD Vero cells was analyzed by WB. (F) PPRV replication in wild-type and METTL3_KD Vero cells was analyzed by virus titration using the TCID_50_ method. Error bars indicate standard deviations; *, *P* < 0.05; **, *P* < 0.01.

METTL3 is a major component of the m^6^A methyltransferase complex involved in creating m^6^A modifications. In order to understand the effects of reduction of METTL3 protein in the host cell on PPRV replication, we generated a stable knockdown clone of the METTL3 gene in Vero cells (METTL3_KD). The stable clones showed lower METTL3 mRNA and protein (Fig. 6A and B). Further, METTL3_KD cells expectedly showed a reduction in m^6^A modifications in the mRNA compared to wild-type (WT) Vero cells (Fig. 6C). Next, to assess the role of host METTL3 on viral replication, both METTL3_KD and WT Vero cells were infected with PPRV. The results indicated that both the mRNA and protein of the PPRV nucleocapsid (N gene) were significantly reduced in the METTL3_KD cells compared to those in the WT Vero cells at different time points (Fig. 6D and E). Further, it was observed that the viral load steadily increased significantly in the WT cells but not in the METTL3_KD Vero cells (Fig. 6F). At all time points (24 hpi, 48 hpi, and 72 hpi), viral replication was significantly lower in the METTL3_KD cells than in the WT Vero cells.

**FIG 7:**
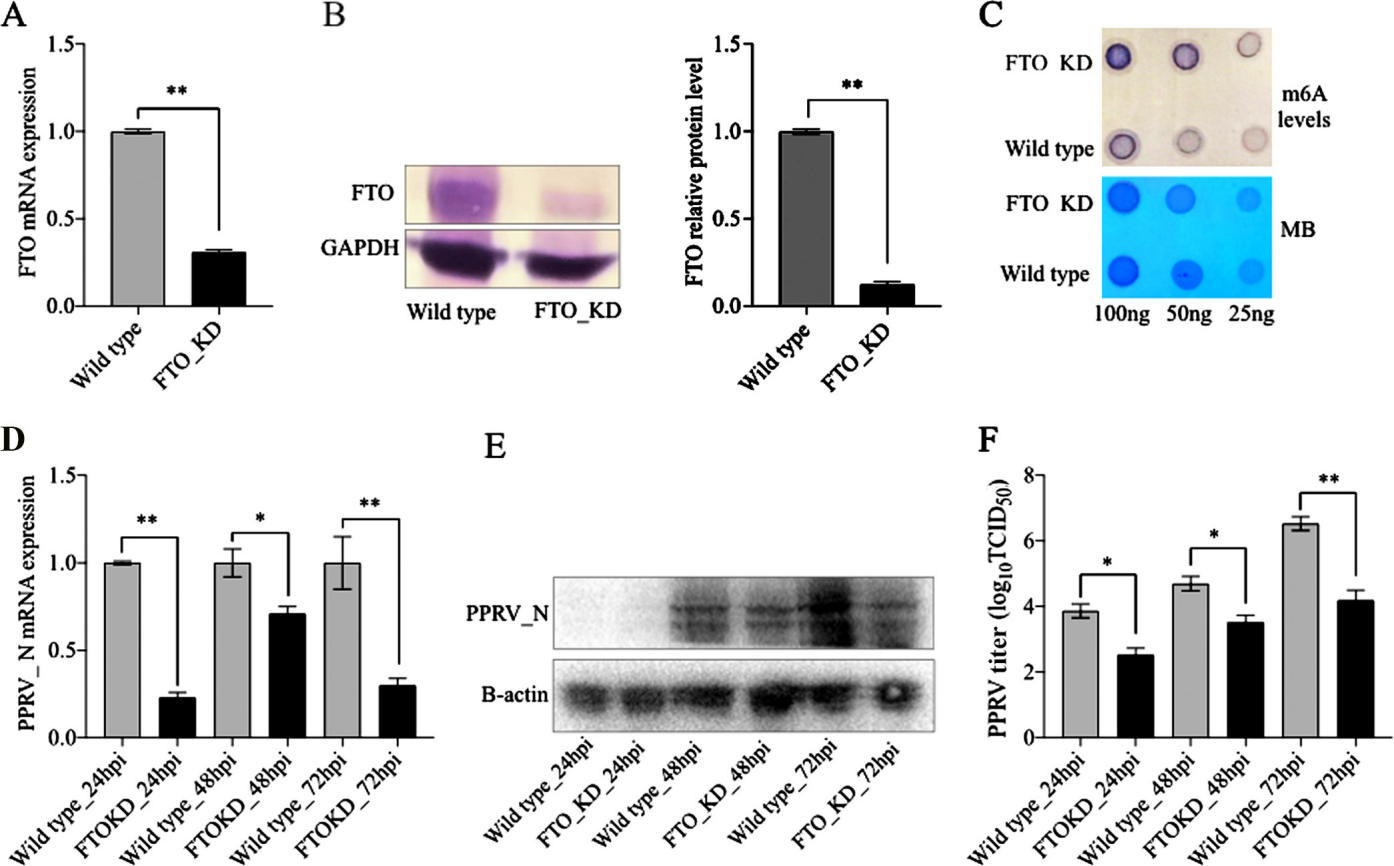
Effect of FTO knockdown on PPRV gene expression and replication. (A) FTO mRNA expression was analyzed in wild-type and FTO stable knockdown (FTO_KD) Vero cells by RT-qPCR. (B) FTO protein level in wild-type and FTO_KD Vero cells was analyzed by WB. (C) Levels of m^6^A modification in mRNA of wild-type and FTO_KD Vero cells were analyzed by dot blot assay, with MB (methylene blue) used as the loading control. (D) PPRV nucleocapsid gene expression in wild-type and FTO_KD Vero cells was analyzed by RT-qPCR. (E) PPRV nucleocapsid (N) protein level in wild-type and FTO_KD Vero cells was analyzed by WB. (F) PPRV replication in wild-type and FTO_KD Vero cells was analyzed by virus titration using the TCID_50_ method. Error bars indicate standard deviations; *, *P* < 0.05; **, *P* < 0.01.

FTO is a demethylase which, along with the ALKBH5, is involved in removing m^6^A modifications. In the present study, we generated a stable knockdown of FTO in Vero cells (FTO_KD). FTO_KD cells showed reduced FTO mRNA and protein (Fig. 7A and B). Due to reduced demethylase activities, as expected, FTO_KD cells showed higher m^6^A levels than the WT Vero cells (Fig. 7C). To assess viral gene expression and replication, next, FTO_KD and WT Vero cells were infected with PPRV. The results indicated that both the mRNA of the PPRV nucleocapsid (N) gene and the viral nucleocapsid protein were lower in the FTO_KD than in the WT Vero cells at different time points of infection (Fig. 7D and E). It was also revealed that the PPRV load increased significantly over a period of time in the WT Vero cells but not in the FTO_KD cells (Fig. 7F). Further, at all time points (24 hpi, 48 hpi, and 72 hpi), viral replication was significantly lower in the FTO_KD cells than in the WT Vero cells. Taken together, these data make it evident that m^6^A methyltransferase (METTL3) and demethylase (FTO) regulate PPRV replication in Vero cells.

### A certain level of m^6^A RNA modification enhances PPRV replication.

**FIG 8:**
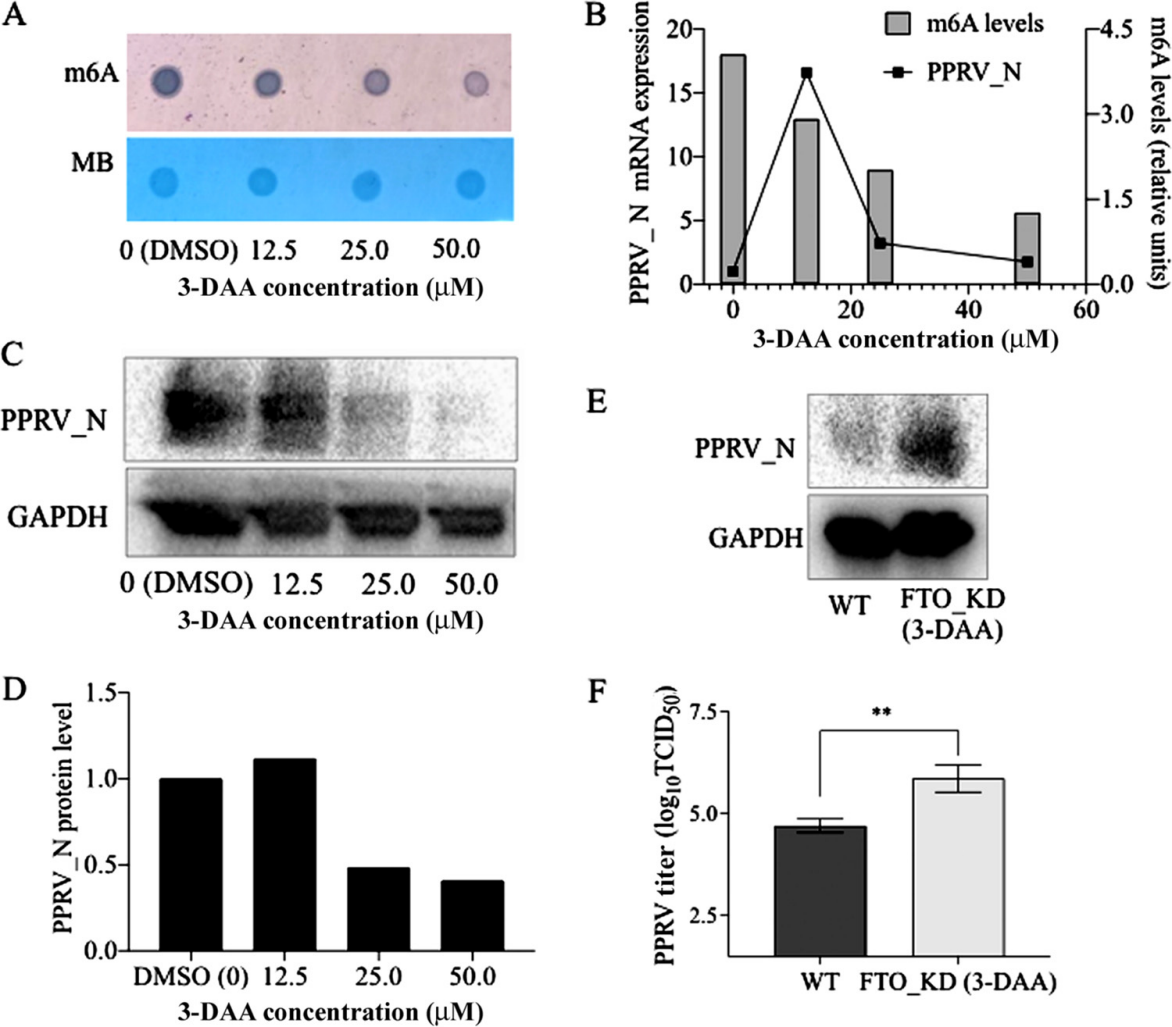
Relationship between m^6^A modification levels and PPRV gene expression and replication. (A) FTO_KD Vero cells (with increased m^6^A modification) treated with different concentrations of 3-DAA show reduced m^6^A modification as analyzed by dot blot assay, with MB (methylene blue) used as the loading control. (B) Comparison of PPRV N gene expression and m^6^A modification levels in FTO_KD cells treated with 3-DAA. (C and D) PPRV N protein level evaluated in FTO_KD cells treated with different concentrations of 3-DAA. (E) PPRV N protein level analyzed in FTO_KD cells treated with 12.5 μM 3-DAA and wild-type (WT) cells. (F) PPRV replication was analyzed in FTO_KD cells treated with 12.5 μM 3-DAA and wild-type (WT) cells using virus titration by TCID_50_ assay. Error bars indicate standard deviations; *, *P* < 0.05; **, *P* < 0.01.

In “m6A methyltransferase and demethylase regulate PPRV replication” (above), we showed that knockdown of methyltransferase (METTL3_KD) and demethylase (FTO_KD) in host cells resulted in decreased PPRV replication. Next, we tested how different degrees of m^6^A modification in host cells affected PPRV replication. For this purpose, we used FTO_KD cells, which had a higher level of m^6^A modifications than the wild-type Vero cells. The m^6^A modifications in the FTO_KD cells were reduced by treating the cells with increasing concentrations (0, 12.5, 25, and 50 μM) of 3-DAA (Fig. 8A). Once the system was validated, FTO_KD cells were treated with different concentrations of 3-DAA for 24 h (sufficient time to reduce the m^6^A levels in these cells) and then infected with PPRV. The m^6^A levels in the mRNA, the PPRV N-gene expression, and viral replication were quantified under each condition. The results confirmed the reduction in m^6^A modifications when FTO_KD cells were treated with different concentrations of 3-DAA (Fig. 8A). Interestingly, there was increased expression of PPRV nucleocapsid protein after treatment with 12.5 μM 3-DAA. However, treatment with higher concentrations of 3-DAA (decreasing the m^6^A modifications) caused a further reduction in the viral gene expression (Fig. 8B). The viral nucleocapsid protein levels showed similar patterns (Fig. 8C and D). We separately subjected FTO_KD cells and WT Vero cells to treatment with 12.5 μM 3-DAA for 24 h, followed by PPRV infection. We found that the viral nucleocapsid protein levels and viral titers were significantly higher in FTO_KD cells treated with 3-DAA (12.5 μM) than in WT Vero cells (Fig. 8E and F). Taken together with the results observed in the previous section, this indicates that neither lower m^6^A modification (as in METTL3_KD) or higher m^6^A modification (as in FTO_KD) support PPRV replication. These data indicate that a certain level of m^6^A modification higher than the basal levels of the wild-type cells is required to facilitate increased PPRV replication.

### m^6^A modification improves PPRV mRNA stability and translation efficiency.

**FIG 9:**
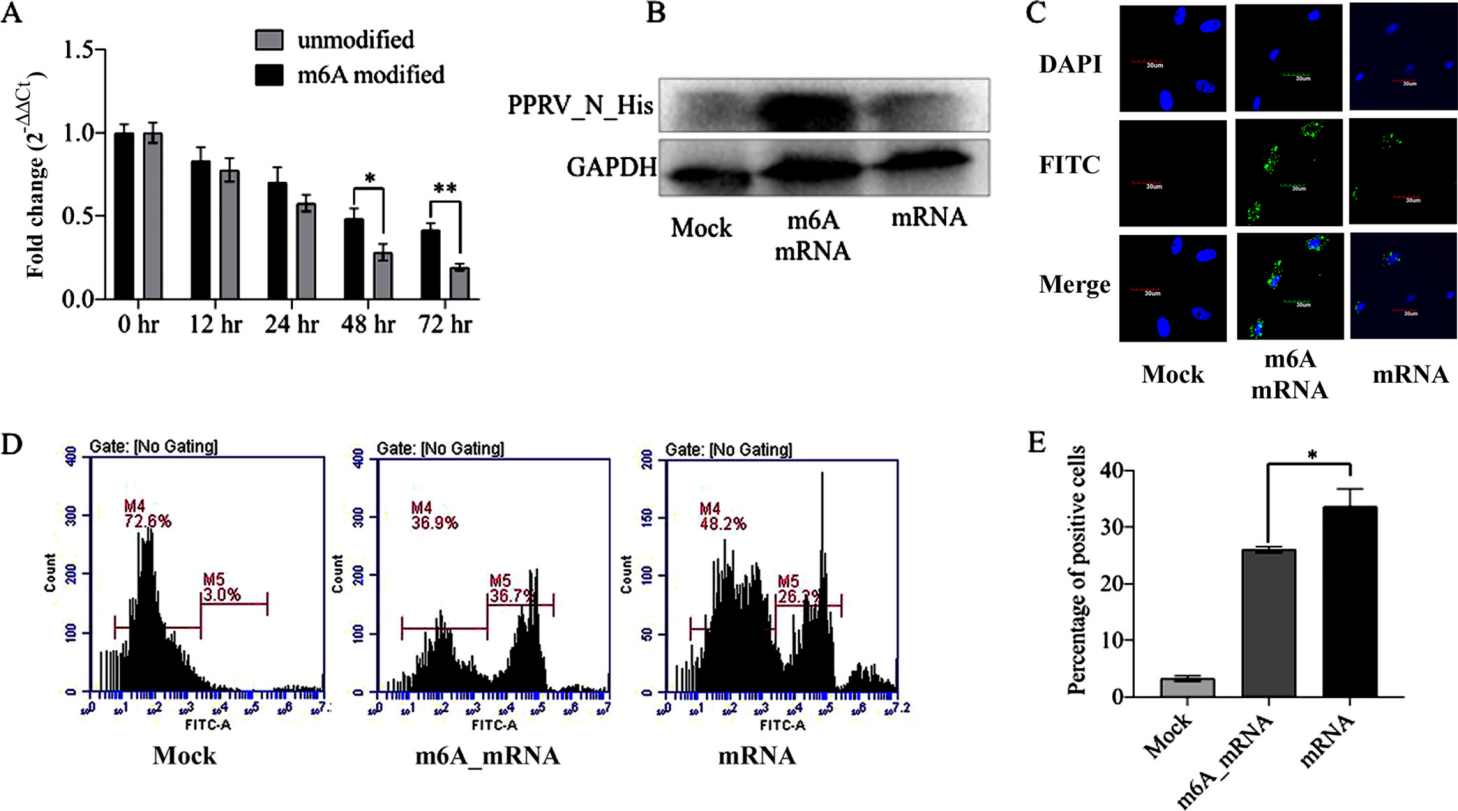
Effect of m^6^A modification on the stability of PPRV nucleocapsid mRNA and efficiency of translation. (A) *In vitro* T7 transcription (IVT) was used to prepare PPRV nucleocapsid mRNA (with or without m^6^A modification). The mRNA was used for transfection of the Vero cells and analyzed for its stability at different time points using RT-qPCR. (B) Vero cells were transfected with PPRV nucleocapsid mRNA with and without m^6^A modification, and the translation efficiency of the mRNA was evaluated by detecting the His-tagged PPRV nucleocapsid protein by WB. (C) His-tagged PPRV nucleocapsid protein was analyzed using immunofluorescence staining of cells transfected with m^6^A-modified and -unmodified PPRV nucleocapsid mRNA. (D and E) Flow cytometric analysis of cells transfected with m^6^A-modified and -unmodified PPRV nucleocapsid mRNA. Error bars indicate standard deviations; *, *P* < 0.05.

We investigated the impact of m^6^A modifications on mRNA stability and translation efficiency by incorporating them with or without m^6^-ATP during *in vitro*-transcribed mRNA synthesis. For *in vitro* transcription, we used a partial PPRV nucleocapsid gene sequence and other essential elements for its translation, along with a His tag. Cultured Vero cells were transfected separately with the m^6^A-modified mRNA and unmodified mRNA. These cells were analyzed for stable mRNA copies that remained at subsequent time points after transfection. The results indicated that the modified mRNA was more stable than the unmodified mRNA from 24 h to 72 h (Fig. 9A). The translation was higher from the m^6^A-modified mRNA than from the unmodified mRNA (Fig. 9B). Similar observations were made about the immunofluorescence staining (Fig. 9C) and flow cytometric (Fig. 9D and E) results. These findings indicate that m^6^A modification facilitates both higher stability and translation efficiency of the viral transcripts.

## DISCUSSION

m^6^A modification of viral RNA was first detected about 4 decades ago (27, 28). However, recent observations have shown the importance of m^6^A modifications in virus-host interactions, which has stimulated interest in finding m^6^A-modified sites in different viruses (29, 30). In the present study, we observed m^6^A modifications in both PPRV genomic RNA and viral mRNAs (tested for the N, P, F, M, and H genes). Previously, m^6^A modifications were reported for a variety of viruses, including HIV, influenza A virus, SV-40, RSV, HSV-1, HMPV, and SARS-CoV-2 (16, 19–25). Earlier, different studies on the same virus showed variations in the m^6^A-modified regions. For example, 14 distinct m^6^A methylation peaks were located in the splicing junctions, coding regions, and noncoding regions of HIV-1 (14). However, later, another group identified only four clusters of m^6^A modifications containing 2 or 3 m^6^A peaks in the HIV-1 genome (16). The variations in the results were likely due to differences in the specificity of the m^6^A antibody used in RNA immunoprecipitation enrichment before next-generation sequencing (MeRIP-Seq). The strength of signals of m^6^A modification in viral RNA can be enhanced by the use of different methods to confirm the detection of m^6^A modifications. Our dot blot, Northern blot, and MeRIP-Seq results showed the presence of m^6^A modification sites in PPRV RNA. Similar methods were used to identify the presence of m^6^A modifications in SARS-CoV-2 and enterovirus 71 (24, 31). It would be of great interest to ascertain the possible positions of modified adenosine bases in viral RNA using the cross-linking and immunoprecipitation (miCLIP) technique.

Viruses can impact host m^6^A modification machinery to create a favorable microenvironment for their replication. We wanted to know whether PPRV infection alters the host m^6^A modification levels and has any effect on the expression of m^6^A writer and eraser proteins. It was found that m^6^A levels in the PPRV-infected host cells decreased initially (24 hpi) but increased at later time points (48 hpi and 72 hpi). The expression patterns of m^6^A writer (METTL3 and WTAP) and eraser (ALKBH5) proteins appeared to support these findings. Previously, it was reported that HIV-1 modulates the dynamics of m^6^A methylation in host T cells. At the active stage of HIV-1 replication (72 hpi), human MT4 CD4^+^ T cells showed significantly higher m^6^A levels than the uninfected control cells (14). Our observations also reflect similar higher levels of m^6^A in PPRV-infected cells at 72 hpi. Interestingly, in Kaposi’s sarcoma-associated herpesvirus (KSHV)-infected cells, the level of m^6^A methylation was increased when latent KSHV was stimulated to undergo lytic replication (13). Taken together, these findings indicate that virus-induced higher m^6^A modification in host cells may facilitate active viral replication.

We also found virus-induced changes in the subcellular localization of methyltransferase (METTL3) and demethylase (FTO). PPRV infection causes redistribution of predominantly nuclear METTL3 to the cytoplasm. After PPRV infection, FTO protein becomes more concentrated in the nucleus. Similar virus-induced changes in the subcellular localization of methyltransferase and demethylase were previously reported in enterovirus-infected Vero cells (31). It has been shown that cells subjected to heat shock display redistribution of m^6^A modification proteins to the cytoplasm to facilitate the translation of stress proteins (32). Viral infection induces a host cell stress response whereby m^6^A modification might favor translation by changes in the subcellular localization of METTL3.

Treatment with 3-deazaadenosine (3-DAA) reduces the formation of S-adenosyl methionine (SAM) and inhibits all types of methylation reactions. Hence, reduction of the levels of m^6^A of mRNA is one of the effects of 3-DAA treatment. We found that 3-DAA treatment decreased m^6^A methylation in the host Vero cells, and it also reduced PPRV gene expression and its replication. The mechanism of host m^6^A reduction is a recent understanding that explains the earlier findings of 3-DAA-mediated antiviral effects against multiple viruses (33–35). This inhibitory mechanism of m^6^A editing by 3-DAA and its effect on the replication of viruses have been further explored in recent studies. It was reported that 3-DAA treatment significantly reduced the viral gene expression and replication of HSV-1 and HIV-1 (23, 36). Our results similarly indicated that reducing m^6^A RNA methylation levels has an inhibitory effect on PPRV gene expression and replication. These findings also corroborate our data showing that knockdown of METTL3 (specific reduction of m^6^A modification levels) caused a reduction in PPRV gene expression and replication.

Initially, it was reported that FTO protein uses m^6^Am (N^6^,2′-O-dimethyladenosine)-modified RNA as its substrate (37). However, later studies clearly indicated that the major substrate for FTO demethylation activity is m^6^A modification of mRNA (38). Meclofenamic acid (MA) was found to specifically inhibit the m^6^A eraser function of FTO, and its competitive inhibition was confirmed through detailed structure-based results (39). Inhibition of the m^6^A eraser function of FTO protein leads to impairment in different biological functions (40, 41). We used MA to understand the effect of increased m^6^A methylation levels in host cells on PPRV replication. MA treatment increased PPRV gene expression and its replication in a dose-dependent manner but only to a certain extent. The results suggest that higher m^6^A than the basal levels of host Vero cells facilitates viral replication.

We took another approach to reducing the major m^6^A machinery proteins to capture their effect on PPRV replication. Accordingly, we generated host cells with stable knockdown of METTL3 and FTO genes. These knockdown clones expectedly exhibited their respective effects on m^6^A modifications. Interestingly, we found reduced PPRV gene expression and replication in both the METTL3 and FTO knockdown conditions. Earlier studies reported that silencing of METTL3 reduced viral replication, whereas silencing FTO increased replication, for viruses such as RSV, HSV-1, SARS-CoV-2, and enterovirus 71 (21, 23–25, 31). However, the direct correlation between the level of m^6^A editing and viral replication was not withstanding the conflicting results reported by some studies. For example, depletion of m^6^A methyltransferases or an m^6^A demethylase respectively increases or decreases viral replication in the case of flaviviruses (18). On the contrary, we found decreased PPRV replication in both METTL3 and FTO knockdown cells. These divergent results may be due to the type of host cells, infection stages, and strategies used by different viruses.

**FIG 10:**
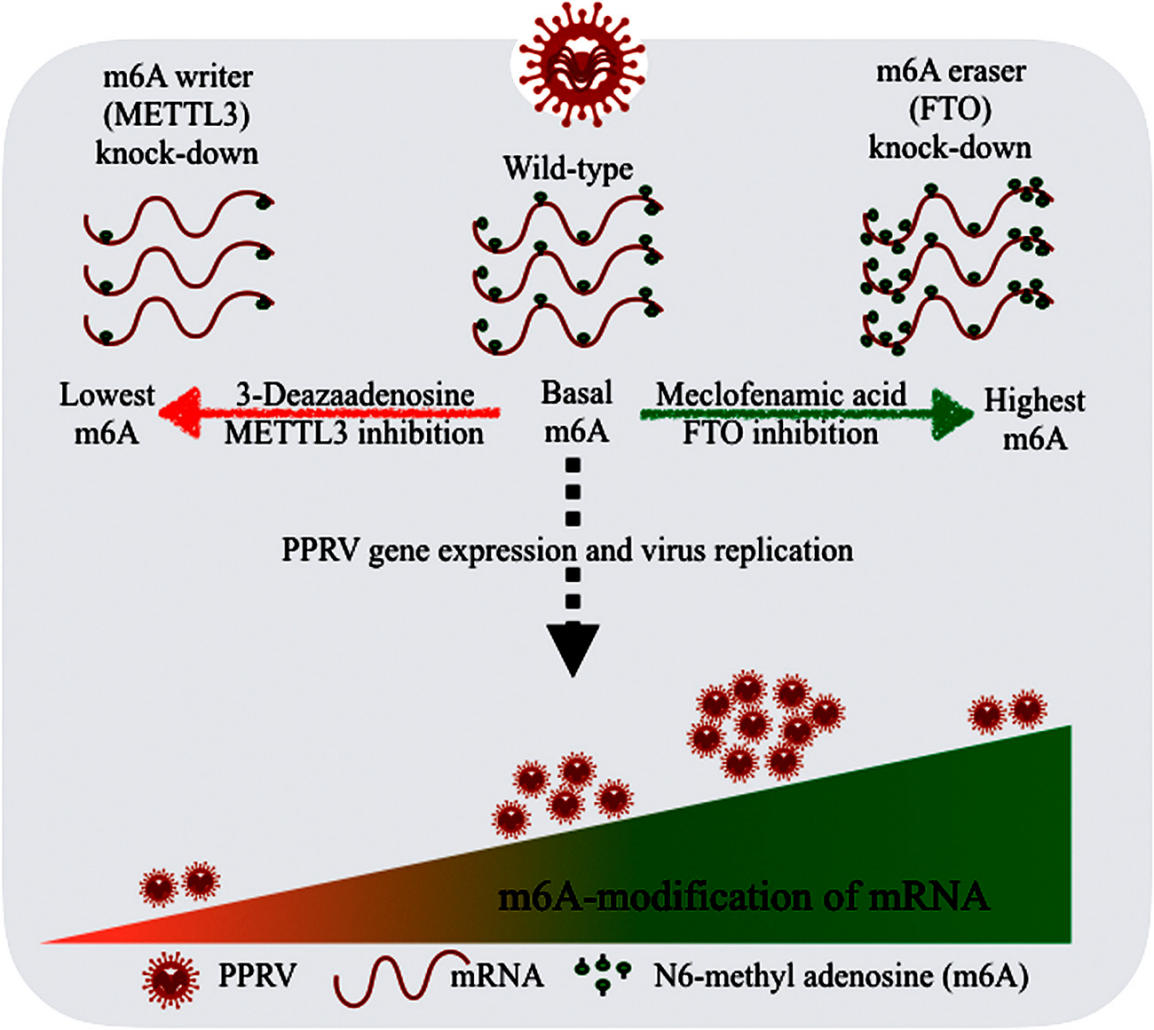
Schematic diagram showing the relationship between m^6^A modification of host Vero cells and PPRV replication. Neither greatly reduced or increased levels of N^6^-methyladenosine (m^6^A) modifications in host cells favored PPRV gene expression and viral replication. The highest PPRV replication requires certain optimal m^6^A modification levels, higher than the basal m^6^A modifications in wild-type Vero cells.

Further, to confirm the relationship between the levels of m^6^A modification and PPRV replication, we treated stable FTO knockdown cells (which had higher m^6^A levels than the wild-type cells) with 3-DAA and analyzed PPRV replication. Specifically, at certain m^6^A levels (achieved by treatment of FTO_KD cells with 12.5 μM 3-DAA), the PPRV gene expression and viral replication were increased. Our results indicate that the maximum viral replication occurs with optimal m^6^A editing, and this phenomenon may be unique for the virus and the host cells involved. The requirement of a specific m^6^A editing level may be essential for the viral RNA stability, specific localization, and translation that determine viral replication (42). Further, successful viral replication is potentially linked to the m^6^A alterations in the host cells (5). In the present study, we created a platform of host cells by stably knocking down major m^6^A machinery proteins, and this platform enabled us to set different levels of m^6^A modifications using small-molecule inhibitors. Our results indicated a relationship between the m^6^A RNA levels of the host cells and PPRV replication (Fig. 10).

Among the different molecular mechanisms that regulate mRNA half-life (stability and degradation), m^6^A modification is considered to play a major role (43). In conjunction with m^6^A-modified sites, YTH domain-containing proteins (YTHDF1, YTHDF2, and YTHDF3) regulate different aspects of the mRNA life cycle (44). The present study assessed the effect of m^6^A modification on the biology of viral mRNA. The results indicate that m^6^A-modified PPRV mRNA had a longer half-life and higher translation efficiency than the unmodified PPRV mRNA. Previously, it was reported that YTHDF2 binds m^6^A sites and recruits adaptor proteins to trigger the degradation of m^6^A-containing mRNA (45, 46). YTHDF3, on the other hand, facilitates the translation of m^6^A-modified mRNA in collaboration with YTHDF1 and influences the YTHDF2-mediated decay process (47). This indicates that viral RNA utilizes m^6^A modification to promote translation and turnover, contributing to viral replication in the host cells.

In summary, the present study revealed that PPRV has m^6^A-modified sites in its RNA and that PPRV affects the dynamics of m^6^A modification in the infected host cells. Reducing the host m^6^A modification levels decreased the PPRV replication, whereas increasing the host m^6^A levels enhanced viral replication up to a certain limit. Further, host cells with stable knockdown of the METTL3 and FTO genes did not completely support PPRV replication compared to the wild-type cells. It was found that both higher and lower levels of m^6^A modification in the host cells may adversely affect viral replication. Higher PPRV gene expression and replication were observed at an optimal level of m^6^A editing in the host cells. Based on these results, we summarized the relationship between host m^6^A levels and PPRV replication (Fig. 10). Further, m^6^A-modified viral transcripts had better stability and translation efficiency compared to unmodified mRNA. Together, these results indicate that the process of m^6^A modification in host cells regulates PPRV replication. These findings contribute to a way forward to regulate viral replication and devise novel antiviral strategies by modulating the host m^6^A modification dynamics.

## MATERIALS AND METHODS

### Cell culture and viruses.

Vero cells (African green monkey kidney cells) were cultured in Dulbecco’s modified Eagle’s medium (12800-017; Gibco) supplemented with 10% fetal bovine serum (1600-044; Gibco) with 5% CO2 at 37°C. Peste des petits ruminants virus (PPRV) (strain Sungri/96) was obtained from the Division of Virology, Mukteshwar, Indian Veterinary Research Institute (IVRI), and was propagated and multiplied in Vero cells. Titration was determined as the 50% tissue culture infective dose (TCID_50_) using the Reed-Muench formula (48).

### m^6^A dot blot analysis.

Supernatant from the PPRV-infected Vero cell culture was collected and clarified to remove cell debris by centrifugation at 8,000 rpm for 10 min. The clarified fluid was filtered and subjected to ultracentrifugation to pellet the virus. RNA was isolated from the purified virus using TRIzol reagent (15596026; Invitrogen). *In vitro* PPRV RNA was transcribed from plasmid DNA template containing an N-gene sequence using the MEGAscript T7 kit (AM1333; Ambion) according to the manufacturer’s instructions, with or without the addition of m^6^-ATP for modified or unmodified IVT RNA. For dot blot analysis, PPRV RNA and *in vitro*-transcribed RNA were adsorbed onto positively charged N+ Hybond membranes (11209299001; Roche), followed by UV cross-linking. Detection of m^6^A modifications was performed using anti-m^6^A antibody (202003; Synaptic Systems). There are always concerns regarding the specificity of anti-m^6^A antibody. However, the anti-m^6^A antibody used in the present study has been widely reported in other previous reports (24). We used anti-rabbit-alkaline phosphatase (AP) secondary antibody with a BCIP (5-bromo-4-chloro-3-indolyl phosphate)-NBT (nitro blue tetrazolium) detection system (B1911; Sigma-Aldrich). Further, the dot blot assay for m^6^A level estimation is a semiquantitative method, and it cannot capture subtle differences in m^6^A levels.

### Methylated RNA immunoprecipitation.

MeRIP was conducted following a previously described protocol (49). Briefly, total RNA (250 μg) was isolated from Vero cells infected with PPRV at 48 hpi. Purification of mRNA from total RNA was performed using Dynabeads oligo(dT)_25_ (61005; Life Technologies) according to the manufacturer’s instructions. The purified mRNA was fragmented using Ambion RNA fragmentation reagents (AM8740; Life Technologies) and repurified with overnight ethanol precipitation. Part of the fragmented RNA was retained as an input control. m^6^A RNA enrichment was performed using the EpiMark N^6^-methyladenosine enrichment kit (E1610S; NEB) following the manufacturer’s instructions. Briefly, m^6^A antibody was attached to protein G magnetic beads by incubation with orbital rotation for 30 min at 4°C. The prepared RNA was bound to the beads coated with m^6^A by incubation with orbital rotation for 1 h at 4°C. After careful washing, the enriched RNA was eluted with elution buffer, followed by cleanup and concentration of the eluted RNA. MeRIP and input control RNA were used for further analysis.

### Northern blotting.

Northern blotting was performed using the DIG Northern starter kit (12039672910; Roche), following the manufacturer’s instructions. Briefly, input RNA and m^6^A-MeRIP RNA were separated on 2% agarose with 2.2-M formaldehyde gels in running buffer (20 mM MOPS [morpholinepropanesulfonic acid], 5 mM sodium acetate, 2 mM EDTA, pH 7.0). The RNAs were transferred to Hybond N+ hybridization membranes (11209299001; Roche) in 20× SSC buffer (3.0 M NaCl, 0.3 M sodium citrate) overnight. The transferred RNA was UV cross-linked to the membrane and hybridized with a DIG-labeled PPRV N-gene probe (100 ng/mL) for 6 h at 68 °C. The membrane was washed 2 times with 2× SSC buffer and 2 times with 0.1× SSC and 0.1% SDS. The membrane was blocked with blocking solution for 30 min at room temperature (RT) and incubated for 30 min in antibody solution. The membrane was washed with washing buffer and equilibrated for 2 to 5 min in detection buffer; the membrane was then placed in CDP-Star solution. Detection signals were developed on a ChemiDoc MP imaging system (Bio-Rad Laboratories, USA).

### MeRIP-Seq and analysis.

MeRIP sequencing of RNA (input transcriptome sequencing [RNA-Seq] and MeRIP-Seq) was performed by preparing libraries using Illumina TruSeq stranded mRNA kits following the manufacturer’s instructions. The samples were sequenced on a HiSeq 2500 instrument in single-read 50-base format. Bioinformatics analysis of the MeRIP-Seq data was carried out following standard methods. Briefly, RNA-Seq reads were aligned to the PPRV genome (GenBank accession no. KF727981) using BWA software, keeping only uniquely mapping reads. Peaks were called using exomePeak, with 20-bp windows to test for statistically significant enrichment in the Immunoprecipitation (IP) relative to the control input RNA with an adjusted (Benjamini-Hochberg [BH]) *P* value cutoff of 0.05. The aligned reads and coverage were visualized using IGV and other features of BEDTools 2.5.

### Western blot analysis.

Proteins were analyzed using the standard Western blot protocol; cell lysates were prepared, after washing the monolayer with ice-cold phosphate-buffered saline (PBS) twice, and harvested into mammalian protein extraction reagent (M-PER; 78503; Thermo Fisher Scientific) containing protease inhibitor (86-331; G Biosciences) for total protein extraction. The protein concentration was measured using a BCA kit (2322; Thermo Scientific). Protein lysate was diluted in 5× Laemmli buffer (ML121; HiMedia), heated at 95°C for 5 min, and immediately placed on ice. Subsequently, proteins were separated on 12% SDS-PAGE and transferred to a nitrocellulose membrane (IEVH85R; Merck). The membranes were blocked with 5% bovine serum albumin (BSA; MB083; HiMedia) for 1 h at RT, followed by overnight incubation with primary antibody at 4°C, and washed 3 times with PBS with Tween 20 (PBST). The membranes were incubated with secondary antibody for 1 h at RT. After 3 washes with PBST, the membranes were incubated with the appropriate substrate (WBLUF0100 or B6404; both from Merck) for developing the signals. The primary antibodies used in our study were as follows: mouse monoclonal antibody against GAPDH (glyceraldehyde-3-phosphate dehydrogenase) (AM4300; Invitrogen), rabbit polyclonal antibody against B-actin (ab8227; Abcam), rabbit monoclonal antibody against METTL3 (15073-1-AP; Proteintech), anti-METTL14 (SAB1104405; Sigma-Aldrich), anti-WTAP (ab155655; Abcam), anti-ALKBH5 (ab69325; Abcam), anti-FTO (ab124892; Abcam), anti-YTHDF1 (17479-1-AP; Proteintech), and mouse monoclonal antibody against PPRV N protein, generated in-house and gifted by R. P. Singh’s lab. The secondary antibodies used in the study were goat anti-mouse IgG and goat anti-rabbit IgG (65-6122; Invitrogen). The band intensities were measured using ImageJ software.

### Double immunofluorescence confocal microscopy.

Vero cells were seeded into six-well plates 1 day before infection at ~50% confluence; then, the cells were infected with PPRV (multiplicity of infection [MOI], 1.0) and incubated for the indicated times. Indirect immunofluorescence assay was performed. In brief, the cells were washed three times with PBS, fixed in 4% paraformaldehyde (R143; G Biosciences) in PBS for 15 min, permeabilized in 0.5% Triton X-100 (MB031-100ML; HiMedia) for 15 min, and blocked in 5% bovine serum albumin (MB083-25G; HiMedia) for 30 min at room temperature. The cells were incubated with primary antibodies, diluted as suggested by the manufacturer, overnight at 4°C; the cells were then washed three times with PBS and stained with the corresponding secondary antibody for 1 h at room temperature. The nuclei were stained with DAPI (D9542; Sigma). The slides were observed under a confocal microscope (FV1000; Olympus).

### Generation of stable knockdown Vero cells.

Lentivirus transduction particles (Mission; SHCLNV; Sigma) expressing short hairpin RNA (shRNA) and specifically targeting the m^6^A writer protein, METTL3 (SCHLNV NM_019852; GPP Web Portal identifier, TRCN0000289812; target sequence, CGTCAGTATCTTGGGCAAGTT), and the m^6^A eraser protein, FTO (SCHLNV; GenBank accession no. NM_001080432; GPP Web Portal identifier, TRCN0000246250; target sequence, TCACCAAGGAGACTGCTATTT), were used for generating stable knockdown Vero cells. Cells (60% to 70% confluent) were transduced with particles following the manufacturer’s instructions. Briefly, after 6 h of incubation, fresh medium was added with puromycin for selection, and the medium was replenished every 3 to 4 days. Individual resistant colonies were picked up and expanded. The clones were tested for knockdown of the target genes (METTL3 and FTO) using RT-qPCR and Western blot assay.

### Reverse transcription-quantitative PCR.

Total RNA was extracted using TRIzol reagent (15596026; Invitrogen). Reverse transcription was performed with 3 μg of total RNA using the RevertAid cDNA synthesis kit (K1622; Thermo Fisher). RT-qPCR was performed using SYBR green QuantiFast PCR master mix (204054; Qiagen) on a CFX Connect real-time system (Bio-Rad).

### Statistical analysis.

Statistical analysis of the RT-qPCR data and other observations was performed using a two-tailed unpaired *t* test using GraphPad Prism software (La Jolla, CA, USA). Data are presented as the means ± standard error of the mean (SEM) (*n* = 3). All experiments were repeated at least three times.

### Data availability.

The MeRIP-Seq data have been submitted to GenBank under the BioProject accession no. PRJNA896352.

## References

[1] Summers WC. 2009. Virus infection, p 546–552. *In* Schaechter M (ed), Encyclopaedia of microbiology, 3rd ed. Academic Press, Cambridge, MA.

[2] Birch EW, Ruggero NA, Covert MW. 2012. Determining host metabolic limitations on viral replication via integrated modelling and experimental perturbation. PLoS Comput Biol 8:e1002746. doi:10.1371/journal.pcbi.1002746. 23093930 PMC3475664

[3] Thaker SK, Ch'ng J, Christofk HR. 2019. Viral hijacking of cellular metabolism. BMC Biol 17:59. doi:10.1186/s12915-019-0678-9. 31319842 PMC6637495

[4] Rampersad S, Tennant P. 2018. Replication and expression strategies of viruses, p 55–82. *In* Tennant P, Fermin G, Foster JE (ed), Viruses: molecular biology, host interactions and applications to biotechnology. Academic Press, Cambridge, MA.

[5] Barranco C. 2020. Viral infection linked to m^6^A alterations in host mRNAs. Nat Rev Mol Cell Biol 21:64–65. doi:10.1038/s41580-019-0202-7. 31853005

[6] Yang Y, Hsu PJ, Chen Y-S, Yang Y-G. 2018. Dynamic transcriptomic m^6^A decoration: writers, erasers, readers and functions in RNA metabolism. Cell Res 28:616–624. doi:10.1038/s41422-018-0040-8. 29789545 PMC5993786

[7] Meyer KD, Jaffrey SR. 2017. Rethinking m^6^A readers, writers, and erasers. Annu Rev Cell Dev Biol 33:319–342. doi:10.1146/annurev-cellbio-100616-060758. 28759256 PMC5963928

[8] Duan H-C, Wang Y, Jia G. 2019. Dynamic and reversible RNA N6-methyladenosine methylation. Wiley Interdiscip Rev RNA 10:e1507. doi:10.1002/wrna.1507. 30252201

[9] Manners O, Baquero-Perez B, Whitehouse A. 2019. m^6^A: widespread regulatory control in virus replication. Biochim Biophys Acta Gene Regul Mech 1862:370–381. doi:10.1016/j.bbagrm.2018.10.015. 30412798 PMC6414752

[10] Gonzales-van Horn SR, Sarnow P. 2017. Making the mark: the role of adenosine modifications in the life cycle of RNA viruses. Cell Host Microbe 21:661–669. doi:10.1016/j.chom.2017.05.008. 28618265 PMC5555051

[11] Gokhale NS, Horner SM. 2017. RNA modifications go viral. PLoS Pathog 13:e1006188. doi:10.1371/journal.ppat.1006188. 28278189 PMC5344520

[12] Kennedy EM, Courtney DG, Tsai K, Cullen BR. 2017. Viral epitranscriptomics. J Virol 91:e02263-16. doi:10.1128/JVI.02263-16. 28250115 PMC5391447

[13] Ye F, Chen ER, Nilsen TW. 2017. Kaposi's sarcoma-associated herpesvirus utilizes and manipulates RNA N^6^-adenosine methylation to promote lytic replication. J Virol 91:e00466-17. doi:10.1128/JVI.00466-17. 28592530 PMC5533915

[14] Lichinchi G, Gao S, Saletore Y, Gonzalez GM, Bansal V, Wang Y, Mason CE, Rana TM. 2016. Dynamics of the human and viral m(6)A RNA methylomes during HIV-1 infection of T cells. Nat Microbiol 1:16011. doi:10.1038/nmicrobiol.2016.11. 27572442 PMC6053355

[15] Kong W, Rivera-Serrano EE, Neidleman JA, Zhu J. 2019. HIV-1 replication benefits from the RNA epitranscriptomic code. J Mol Biol 431:5032–5038. doi:10.1016/j.jmb.2019.09.021. 31626810 PMC6953616

[16] Kennedy EM, Bogerd HP, Kornepati AV, Kang D, Ghoshal D, Marshall JB, Poling BC, Tsai K, Gokhale NS, Horner SM, Cullen BR. 2016. Posttranscriptional m^6^A editing of HIV-1 mRNAs enhances viral gene expression. Cell Host Microbe 19:675–685. doi:10.1016/j.chom.2016.04.002. 27117054 PMC4867121

[17] Courtney DG, Chalem A, Bogerd HP, Law BA, Kennedy EM, Holley CL, Cullen BR. 2019. Extensive epitranscriptomic methylation of A and C residues on murine leukemia virus transcripts enhances viral gene expression. mBio 10:e01209-19. doi:10.1128/mBio.01209-19. 31186331 PMC6561033

[18] Gokhale NS, McIntyre ABR, Mattocks MD, Holley CL, Lazear HM, Mason CE, Horner SM. 2020. Altered m^6^A modification of specific cellular transcripts affects Flaviviridae infection. Mol Cell 77:542–555.e8. doi:10.1016/j.molcel.2019.11.007. 31810760 PMC7007864

[19] Courtney DG, Kennedy EM, Dumm RE, Bogerd HP, Tsai K, Heaton NS, Cullen BR. 2017. Epitranscriptomic enhancement of influenza A virus gene expression and replication. Cell Host Microbe 22:377–386.e5. doi:10.1016/j.chom.2017.08.004. 28910636 PMC5615858

[20] Tsai K, Courtney DG, Cullen BR. 2018. Addition of m^6^A to SV40 late mRNAs enhances viral structural gene expression and replication. PLoS Pathog 14:e1006919. doi:10.1371/journal.ppat.1006919. 29447282 PMC5831754

[21] Xue M, Zhao BS, Zhang Z, Lu M, Harder O, Chen P, Lu Z, Li A, Ma Y, Xu Y, Liang X, Zhou J, Niewiesk S, Peeples ME, He C, Li J. 2019. Viral N^6^-methyladenosine upregulates replication and pathogenesis of human respiratory syncytial virus. Nat Commun 10:4595. doi:10.1038/s41467-019-12504-y. 31597913 PMC6785563

[22] Lu M, Zhang Z, Xue M, Zhao BS, Harder O, Li A, Liang X, Gao TZ, Xu Y, Zhou J, Feng Z, Niewiesk S, Peeples ME, He C, Li J. 2020. N^6^-methyladenosine modification enables viral RNA to escape recognition by RNA sensor RIG-I. Nat Microbiol 5:584–598. doi:10.1038/s41564-019-0653-9. 32015498 PMC7137398

[23] Feng Z, Zhou F, Tan M, Wang T, Chen Y, Xu W, Li B, Wang X, Deng X, He M-L. 2021. Targeting m^6^A modification inhibits herpes virus 1 infection. Genes Dis 9:1114–1128. doi:10.1016/j.gendis.2021.02.004. 35685469 PMC9170584

[24] Zhang X, Hao H, Ma L, Zhang Y, Hu X, Chen Z, Liu D, Yuan J, Hu Z, Guan W. 2021. Methyltransferase-like 3 modulates severe acute respiratory syndrome coronavirus-2 RNA N^6^-methyladenosine modification and replication. mBio 12:e0106721. doi:10.1128/mBio.01067-21. 34225491 PMC8437041

[25] Burgess HM, Depledge DP, Thompson L, Srinivas KP, Grande RC, Vink EI, Abebe JS, Blackaby WP, Hendrick A, Albertella MR, Kouzarides T, Stapleford KA, Wilson AC, Mohr I. 2021. Targeting the m^6^A RNA modification pathway blocks SARS-CoV-2 and HCoV-OC43 replication. Genes Dev 35:1005–1019. doi:10.1101/gad.348320.121. 34168039 PMC8247602

[26] Tsai K, Bogerd HP, Kennedy EM, Emery A, Swanstrom R, Cullen BR. 2021. Epitranscriptomic addition of m^6^A regulates HIV-1 RNA stability and alternative splicing. Genes Dev 35:992–1004. doi:10.1101/gad.348508.121. 34140354 PMC8247604

[27] Moss B, Gershowitz A, Stringer JR, Holland LE, Wagner EK. 1977. 5'-Terminal and internal methylated nucleosides in herpes simplex virus type 1 mRNA. J Virol 23:234–239. doi:10.1128/JVI.23.2.234-239.1977. 196108 PMC515825

[28] Canaani D, Kahana C, Lavi S, Groner Y. 1979. Identification and mapping of N^6^-methyladenosine containing sequences in simian virus 40 RNA. Nucleic Acids Res 6:2879–2899. doi:10.1093/nar/6.8.2879. 223130 PMC327900

[29] Brocard M, Ruggieri A, Locker N. 2017. m^6^A RNA methylation, a new hallmark in virus-host interactions. J Gen Virol 98:2207–2214. doi:10.1099/jgv.0.000910. 28869001

[30] Imam H, Kim GW, Siddiqui A. 2020. Epitranscriptomic (N^6^-methyladenosine) modification of viral RNA and virus-host interactions. Front Cell Infect Microbiol 10:584283. doi:10.3389/fcimb.2020.584283. 33330128 PMC7732492

[31] Hao H, Hao S, Chen H, Chen Z, Zhang Y, Wang J, Wang H, Zhang B, Qiu J, Deng F, Guan W. 2019. N^6^-methyladenosine modification and METTL3 modulate enterovirus 71 replication. Nucleic Acids Res 47:362–374. doi:10.1093/nar/gky1007. 30364964 PMC6326802

[32] Zhou J, Wan J, Gao X, Zhang X, Jaffrey SR, Qian SB. 2015. Dynamic m^6^A mRNA methylation directs translational control of heat shock response. Nature 526:591–594. doi:10.1038/nature15377. 26458103 PMC4851248

[33] de Clercq E, Montgomery JA. 1983. Broad-spectrum antiviral activity of the carbocyclic analog of 3-deazaadenosine. Antiviral Res 3:17–24. doi:10.1016/0166-3542(83)90011-6. 6307139

[34] Fischer AA, Müller K, Scholtissek C. 1990. Specific inhibition of the synthesis of influenza virus late proteins and stimulation of early, M2, and NS2 protein synthesis by 3-deazaadenosine. Virology 177:523–531. doi:10.1016/0042-6822(90)90517-u. 2142557

[35] Flexner CW, Hildreth JE, Kuncl RW, Drachman DB. 1992. 3-Deaza-adenosine and inhibition of HIV. Lancet 339:438. doi:10.1016/0140-6736(92)90133-n. 1346708

[36] Tirumuru N, Zhao BS, Lu W, Lu Z, He C, Wu L. 2016. N^6^-methyladenosine of HIV-1 RNA regulates viral infection and HIV-1 Gag protein expression. Elife 5:e15528. doi:10.7554/eLife.15528. 27371828 PMC4961459

[37] Mauer J, Luo X, Blanjoie A, Jiao X, Grozhik AV, Patil DP, Linder B, Pickering BF, Vasseur JJ, Chen Q, Gross SS, Elemento O, Debart F, Kiledjian M, Jaffrey SR. 2017. Reversible methylation of m6Am in the 5′ cap controls mRNA stability. Nature 541:371–375. doi:10.1038/nature21022. 28002401 PMC5513158

[38] Wei J, Liu F, Lu Z, Fei Q, Ai Y, He PC, Shi H, Cui X, Su R, Klungland A, Jia G, Chen J, He C. 2018. Differential m^6^A, m^6^A_m_, and m^1^A demethylation mediated by FTO in the cell nucleus and cytoplasm. Mol Cell 71:973–985.e5. doi:10.1016/j.molcel.2018.08.011. 30197295 PMC6151148

[39] Huang Y, Yan J, Li Q, Li J, Gong S, Zhou H, Gan J, Jiang H, Jia G-F, Luo C, Yang C-G. 2015. Meclofenamic acid selectively inhibits FTO demethylation of m^6^A over ALKBH5. Nucleic Acids Res 43:373–384. doi:10.1093/nar/gku1276. 25452335 PMC4288171

[40] Zhou P, Wu M, Ye C, Xu Q, Wang L. 2019. Meclofenamic acid promotes cisplatin-induced acute kidney injury by inhibiting fat mass and obesity-associated protein-mediated m^6^A abrogation in RNA. J Biol Chem 294:16908–16917. doi:10.1074/jbc.RA119.011009. 31578283 PMC6851328

[41] Huff S, Tiwari SK, Gonzalez GM, Wang Y, Rana TM. 2021. m^6^A-RNA demethylase FTO inhibitors impair self-renewal in glioblastoma stem cells. ACS Chem Biol 16:324–333. doi:10.1021/acschembio.0c00841. 33412003 PMC7901021

[42] Williams GD, Gokhale NS, Horner SM. 2019. Regulation of viral infection by the RNA modification N^6^-methyladenosine. Annu Rev Virol 6:235–253. doi:10.1146/annurev-virology-092818-015559. 31283446 PMC6884077

[43] Lee Y, Choe J, Park OH, Kim YK. 2020. Molecular mechanisms driving mRNA degradation by m^6^A modification. Trends Genet 36:177–188. doi:10.1016/j.tig.2019.12.007. 31964509

[44] Liao S, Sun H, Xu C. 2018. YTH domain: a family of N^6^-methyladenosine (m^6^A) readers. Genomics Proteomics Bioinformatics 16:99–107. doi:10.1016/j.gpb.2018.04.002. 29715522 PMC6112328

[45] Wang X, Lu Z, Gomez A, Hon GC, Yue Y, Han D, Fu Y, Parisien M, Dai Q, Jia G, Ren B, Pan T, He C. 2014. N^6^-methyladenosine-dependent regulation of messenger RNA stability. Nature 505:117–120. doi:10.1038/nature12730. 24284625 PMC3877715

[46] Hou G, Zhao X, Li L, Yang Q, Liu X, Huang C, Lu R, Chen R, Wang Y, Jiang B, Yu J. 2021. SUMOylation of YTHDF2 promotes mRNA degradation and cancer progression by increasing its binding affinity with m^6^A-modified mRNAs. Nucleic Acids Res 49:2859–2877. doi:10.1093/nar/gkab065. 33577677 PMC7969013

[47] Shi H, Wang X, Lu Z, Zhao BS, Ma H, Hsu PJ, Liu C, He C. 2017. YTHDF3 facilitates translation and decay of N^6^-methyladenosine-modified RNA. Cell Res 27:315–328. doi:10.1038/cr.2017.15. 28106072 PMC5339834

[48] Reed LJ, Muench H. 1938. A simple method of estimating fifty per cent endpoints. Am J Hyg 27:493–497. doi:10.1093/oxfordjournals.aje.a118408.

[49] Dominissini D, Moshitch-Moshkovitz S, Amariglio N, Rechavi G. 2015. Transcriptome-wide mapping of N^6^-methyladenosine by m^6^A-Seq. Methods Enzymol 560:131–147. doi:10.1016/bs.mie.2015.03.001. 26253969

