# Supplementary material for: N6-Methyladenosine RNA Modification in Host Cells Regulates Peste des Petits Ruminants Virus Replication
Source: Microbiol Spectr. 2023 Feb 14;11(2):e02666-22. doi: 10.1128/spectrum.02666-22 (PMC10101086; doi:10.1128/spectrum.02666-22)
Supplement: Supplemental file 1 — Supplemental material. Download spectrum.02666-22-s0001.pdf, PDF file, 0.1 MB [file spectrum.02666-22-s0001.pdf]

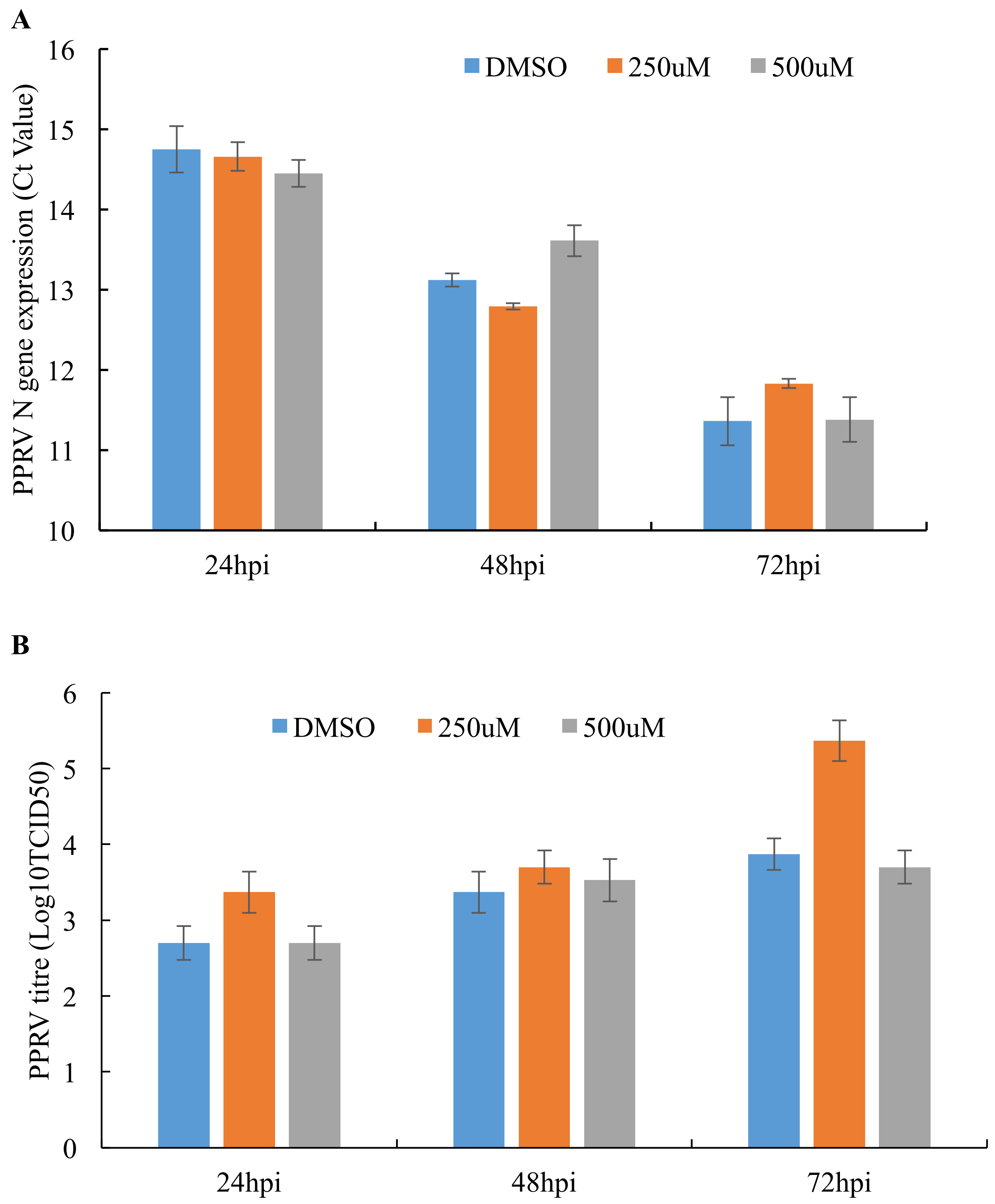

Fig S1: Treatment of host Vero cells with COX enzyme inhibitor Indomethacin does not affect PPRV gene expression and replication. A) PPRV N gene expression was evaluated using qRT-PCR. The comparison was made between control (DMSO) and indomethacin treated (250uM and 500uM) Vero cells at different time points after PPRV infection. B) The PPRV titre was evaluated using TCID50 method. The comparison was made between control (DMSO) and indomethacin treated (250uM and 500uM) Vero cells at different time points after PPRV infection. Error bars indicate standard deviations and the differences were non-significant as measured at $P < 0.01$
